# Re-Expansion Pulmonary Edema as a Life-Threatening Complication in Massive, Long-Standing Pneumothorax: A Case Series and Literature Review

**DOI:** 10.3390/jcm13092667

**Published:** 2024-05-02

**Authors:** Giacomo Cusumano, Luigi La Via, Alberto Terminella, Massimiliano Sorbello

**Affiliations:** 1 Department of General Thoracic Surgery, Azienda Ospedaliero Universitaria Policlinico “G.Rodolico-San Marco”, 95123 Catania, Italy; giacomare55@hotmail.com (G.C.); a.terminella@ao-ve.it (A.T.); 2 Department of Anesthesia and Intensive Care, Azienda Ospedaliero Universitaria Policlinico “G.Rodolico-San Marco”, 95123 Catania, Italy; 3 UOC Anestesia e Rianimazione, Giovanni Paolo II Hospital, 97100 Ragusa, Italy; maxsorbello@gmail.com

**Keywords:** oedema, ventilation, oxygen, X-ray, CT scan

## Abstract

Re-expansion pulmonary edema is a rare and potentially life-threatening complication that can occur after the rapid re-expansion of a collapsed lung due to pneumothorax or pleural effusion. It has a multifactorial pathogenesis, and risk factors for re-expansion pulmonary edema, such as chronic lung collapse, rapid re-expansion, and changes in pulmonary vascular permeability, have been identified. Clinical manifestations vary, ranging from almost asymptomatic to a rapidly fatal condition, and its incidence may be more common and less fatal than previously believed. The literature emphasizes the importance of early recognition and management to ensure favorable outcomes. However, there is ongoing debate regarding the indications for ventilatory support and the timing of non-invasive or invasive ventilation. Herein, we report a case series of three paradigmatic examples of massive re-expansion pulmonary edema occurring over a period of 10 years in our institution among a population of 815 patients with spontaneous pneumothorax. We also conducted a literature review on re-expansion pulmonary edema, with a particular focus on diagnosis and management. In each case, despite initially normal clinical parameters, severe respiratory distress developed following the insertion of a thoracic drainage tube for a massive spontaneous pneumothorax. Two patients required High-Flow Nasal Oxygen, and one was addressed to intensive management, including CPAP. In all cases, the patient’s outcome was optimal.

## 1. Introduction

A primary spontaneous pneumothorax is an abnormal accumulation of air in the pleural cavity. The most common cause of primary spontaneous pneumothorax is the rupture of subpleural blebs. A larger pneumothorax is usually treated by drainage with a chest tube. Surgical treatment is only indicated in cases of recurrent or refractory pneumothorax [1]. Pneumothorax can lead to a number of complications, some of which can be life-threatening. These include respiratory failure or respiratory distress, pulmonary edema after the treatment of pneumothorax, pneumohemothorax, pneumopericardium, pneumoperitoneum, bronchopulmonary fistula, and myocardial infarction [2]. Unilateral re-expansion pulmonary edema is a rare complication resulting from the rapid expansion of the lung following pleural drainage for pneumothorax or pleural effusion. The first description of re-expansion pulmonary edema dates back to 1853 [3], with subsequent reports in the early 1900s noting “albuminous expectoration” as a symptom [4,5]. While this condition is recognized and associated with mortality in severe cases, its actual incidence, including asymptomatic occurrences, may be much more common than previously thought [6]. The condition appears to occur more frequently in young males, particularly following thoracentesis, or pneumothorax [7], with a higher risk observed in those aged between 20 and 39 years [8]. The pathogenesis of re-expansion pulmonary edema is multifactorial and involves various risk factors. These factors include chronic lung collapse, rapid re-expansion, and alterations in pulmonary vascular permeability. Additionally, observations following large-volume thoracentesis suggest the potential involvement of mechanisms related to ischemia/reperfusion injury and increased capillary permeability in the development of re-expansion pulmonary edema [9,10]. The clinical presentation of re-expansion pulmonary edema spans from being clinically asymptomatic to manifesting cardiovascular shock, although the most severe forms are quite rare. There are only a few cases in the literature; thus, various forms of treatment have been reported. The aim of this study is to report our experience in the management of the cases of massive re-expansion pulmonary edema that occurred in our institution over a period of 10 years and among a population of 815 patients presenting with spontaneous pneumothorax. Additionally, we provided the results of a literature review on the pathophysiology and risk factors of the re-expansion pulmonary edema, along with its clinical and radiological features and management, with a particular focus on indications for ventilatory support and the timing for non-invasive or invasive ventilation.

## 2. Materials and Methods

This study aims to assess the incidence and characteristics of re-expansion pulmonary edema, which occurred at Policlinico “G.Rodolico-San Marco” Hospital in Catania, Italy, from January 2014 to January 2024, among a population of 815 spontaneous pneumothorax cases. Over a 10-year period, we observed 3 symptomatic cases of re-expansion pulmonary edema. All cases shared common features, including a delayed diagnosis of large pneumothorax with symptoms persisting for at least 5 days before the diagnosis. In every case, chest X-ray control immediately after chest drain placement showed near-normal findings, yet massive re-expansion pulmonary edema developed clinically and radiologically rapidly within 4 h after chest drain placement. Respiratory support involves High-Flow Nasal Oxygen (HFNO) and Continuous Positive Airway Pressure (CPAP), leading to the resolution of the clinical picture in every case. The first case, detailed below, required intensive management, including HFNO and CPAP, while the remaining two patients achieved resolution with High-Flow Nasal Oxygen alone.

### Literature Search

We also performed a comprehensive narrative review of the literature on the databases PubMed, Scopus, Cochrane, and EMBASE up to 15 February 2024 in order to summarize the current knowledge on re-expansion pulmonary edema, including its pathogenesis, risk factors, clinical and radiological features, and management. Only articles in English were considered. No other restrictions were applied. The search combined the following groups of terms. The first group included the words “oedema” OR “edema” OR “re-expansion” OR “expansion”. The second group included “pulmonary” OR “lung” OR “pneumothorax” OR “PNX” OR “chest” OR “drainage”. The search and the following screening were performed by G.C. and L.L.V. independently in order to find all the relevant articles. Discordances were resolved by involving a third author (M.S.).

## 3. Cases Description

### 3.1. Case 1

**Figure 1:**
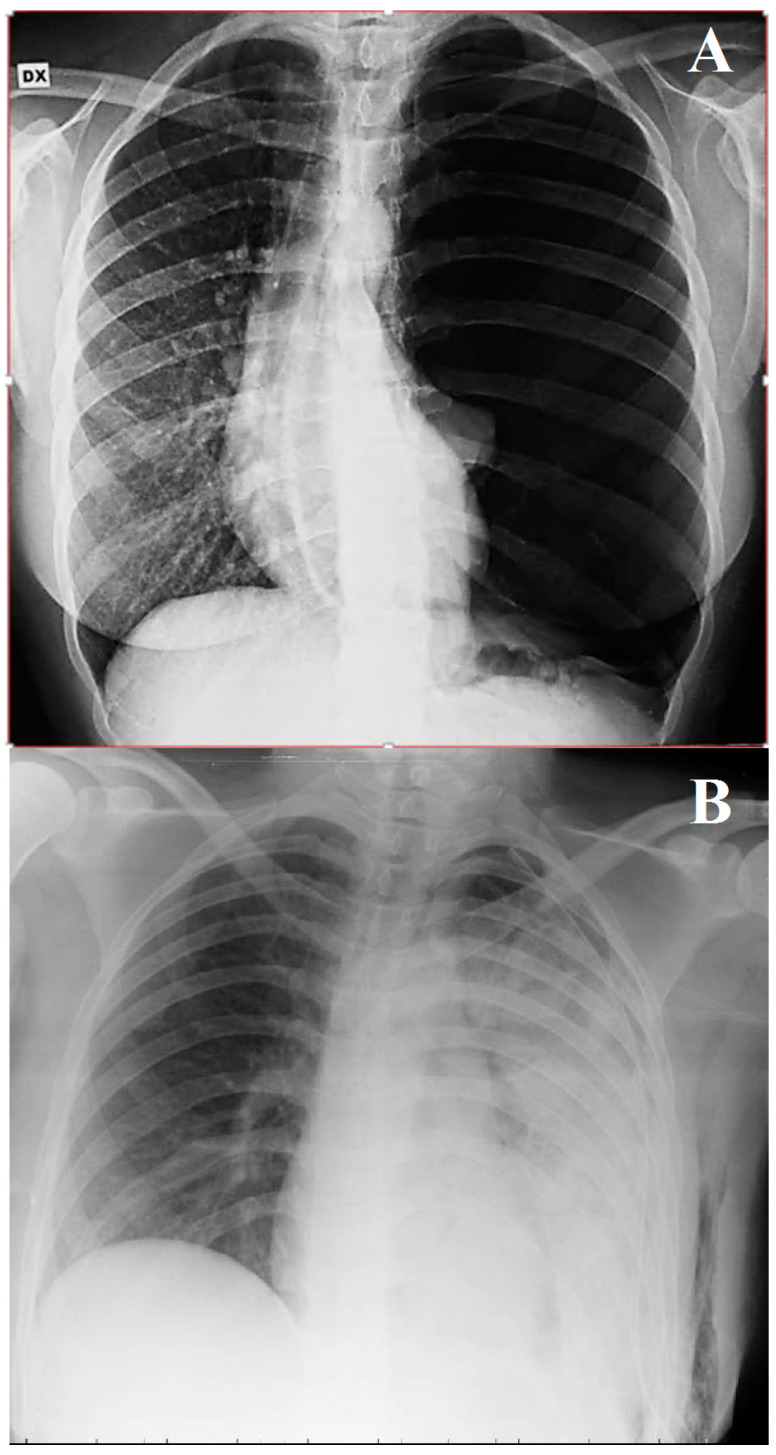
(**A**,**B**) Chest X–ray—(**A**) before drainage; (**B**) edema after re-expansion.

A 29-year-old female volleyball player was admitted to the emergency room for left massive pneumothorax. She reported experiencing mild left chest pain that had started a week earlier after a plane flight. She was a non-smoker, and she did not report any other medical conditions or drug use except for the contraceptive pill. The hospital visit was prompted by exertional dyspnea, particularly during sporting activities, and nonspecific left chest pain. Clinical examination and a chest X-ray revealed a complete collapse of the left lung, although the patient’s vital signs were generally within normal limits, with a room-air pulse oximetry reading of 97% at rest. In the emergency room, a chest drain was placed at the left 6th intercostal space on the middle axillary line, and a negative pressure of 20 cm H_2_O through a two-cambers system was routinely applied to the pleural space. Subsequently, chest X-ray confirmed the complete expansion of the lung, and the patient was then transferred to the thoracic surgery unit for further care. Approximately 2 h later, the patient showed acute respiratory distress, accompanied by coughing with foamy secretion, tachypnea (44 breaths/min), and tachycardia (120 beats/min), while their blood pressure remained within normal range. Upon physical examination, the patient was distressed, sweaty, and agitated but remained conscious. Wet rales were prominently audible throughout the entire left chest on auscultation. The chest drain worked normally with minimal air leakage. Following this, a chest X-ray showed unilateral pulmonary edema associated with re-expansion (Figure 1).

**Figure 2:**
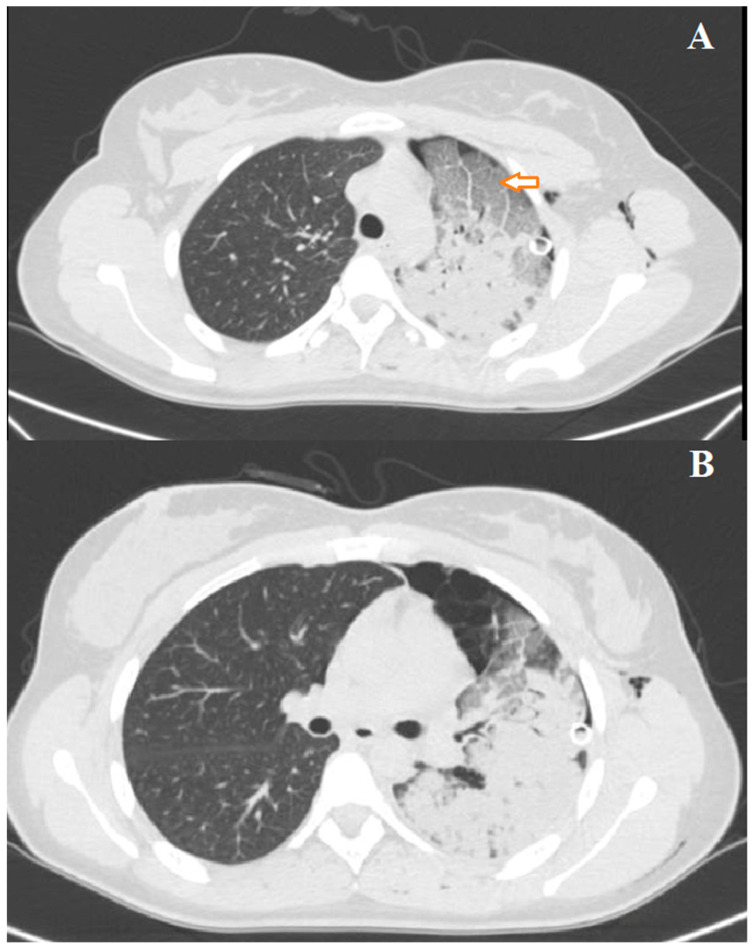
(**A**,**B**) Chest CT scan at presentation of left re-expansion edema; the arrow shows alveolar filling.

The patient was managed into the thoracic surgery unit. Arterial blood gas analysis revealed severe hypoxia with mild hypercapnia (pH: 7.38; partial pressure of carbon dioxide [PCO_2_]: 52 mm Hg; partial pressure of oxygen [PO_2_] mm Hg: 49; and bicarbonate: 24 mEq/L). Oxygen support was given with a venturi mask (FiO_2_ 60%). Diuretics, bronchodilators, and steroids were infused. Despite medical and oxygen support, there was no improvement in the patient’s condition, and arterial blood gas analysis showed the persistence of severe respiratory failure. Oxygen support was then implemented using HFNO, 50 L/min, and 60% FiO_2_. A chest CT scan was also performed to rule out pulmonary embolism. On the contrary, it confirmed the presence of unilateral re-expansion edema with alveolar filling, consolidative areas in the upper and lower left lobes, and a region of bullous emphysema at the anterior and lingular segments of the left upper lobe (Figure 2A,B).

**Figure 3:**
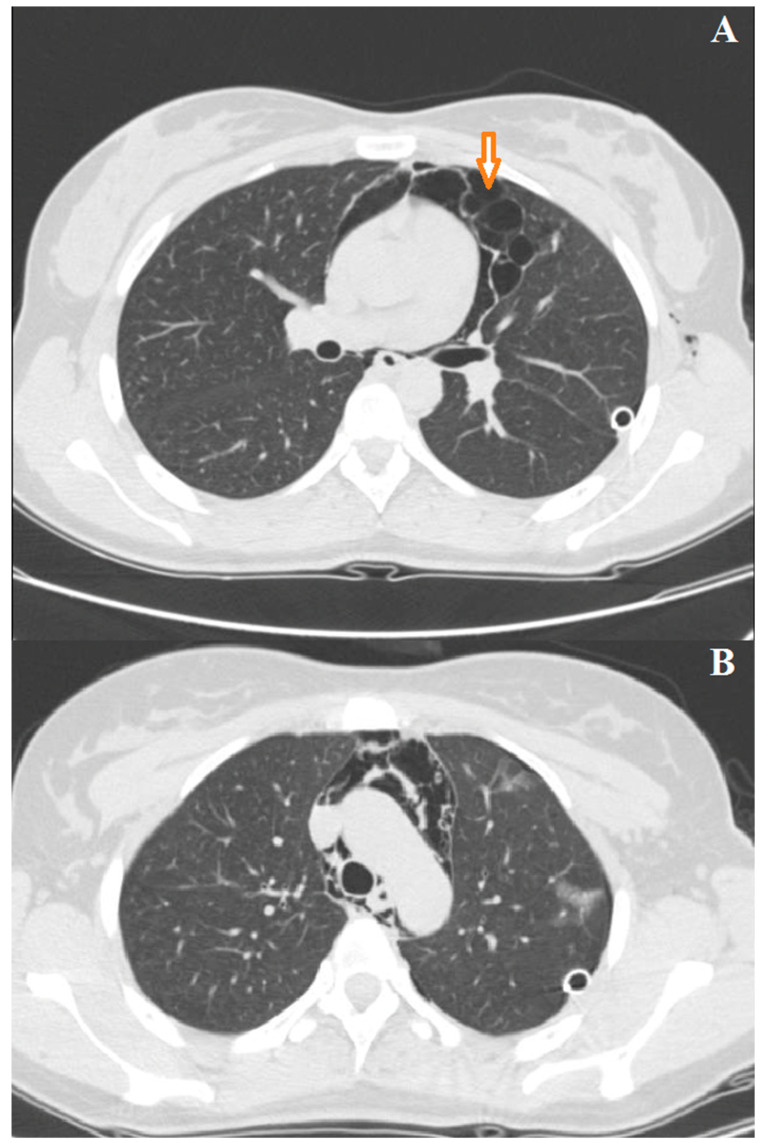
(**A**,**B**) Chest CT scan at resolution of left re-expansion edema; the arrow indicates bullous emphysema.

The patient did not tolerate HFNO, prompting the placement of the CPAP mask with a positive end-expiratory pressure (PEEP) of 10 cmH_2_O. Additionally, the patient was mildly sedated with a midazolam infusion and underwent multiple cycles of respiratory physiotherapy. After 24 h, the patient exhibited gradual recovery, and CPAP was discontinued. A cycle of HFNO (40 L/min, FiO_2_ 50%) was administered for two further days, followed by a de-escalation of O_2_ support with nasal cannulas of 4 L/min. Diuretic and steroidal therapy was maintained for 4 days and gradually tapered off. A follow-up chest CT scan control conducted 4 days later revealed the resolution of edema and lung consolidations with residual pneumothorax and pneumomediastinum (Figure 3A,B).

Finally, seven days later, the patient underwent thoracoscopic apicectomy and mechanical pleurodesis in order to treat persistent pneumothorax. The postoperative period was uneventful, and the patient was discharged 4 days after surgery.

### 3.2. Case 2

**Figure 4:**
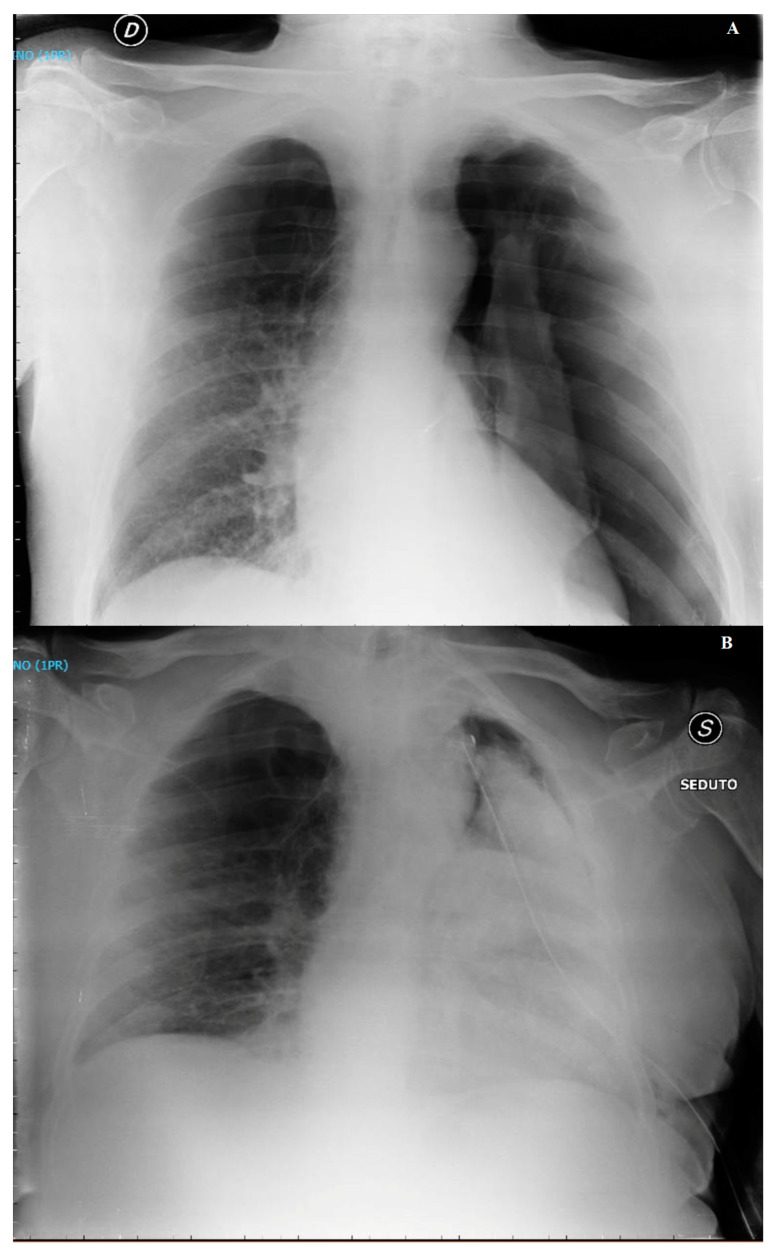
(**A**) Chest X-ray, left massive pneumothorax; (**B**) chest X-ray, left re-expansion edema 6 h after chest tube positioning.

A 69-year-old man with COPD, a former smoker, reported worsening respiratory symptoms for 6 days. A general practitioner prescribed antibiotics and inhaler therapy, suspecting COPD exacerbation. Subsequently, the patient was admitted to the hospital due to the occurrence of respiratory failure. In the emergency room, a tension left pneumothorax was diagnosed and promptly treated with chest drain placement (Figure 4A).

After a temporary clinical improvement, the patient complained of severe dyspnea, coughing, tachypnea, profuse sweating, and tachycardia with persistent air loss from the chest drain. Blood gas analysis revealed hypoxia with pH 7.42; PaCO_2_ = 36 mmHg; PaO_2_ = 54 mmHg; bicarbonate = 26 mM; and SaO_2_ 88% in ambient air. Oxygen therapy with O_2_ nasal cannula at 4 L/min, diuretics, bronchodilators, and steroids were administered. A chest X-ray (Figure 4B) and CT scan confirmed left re-expansion pulmonary edema with alveolar filling and a consolidative area. The patient was then supported with HFNO (flow 60 L/min, FiO_2_ 50%) for 3 days. Radiological controls at 6 days showed the resolution of re-expansion pulmonary edema. Seven days later, the patient underwent thoracoscopic apicectomy and mechanical pleurodesis due to persistent pneumothorax. The recovery after surgery was uneventful, and the patient was discharged five days after surgery.

### 3.3. Case 3

**Figure 5:**
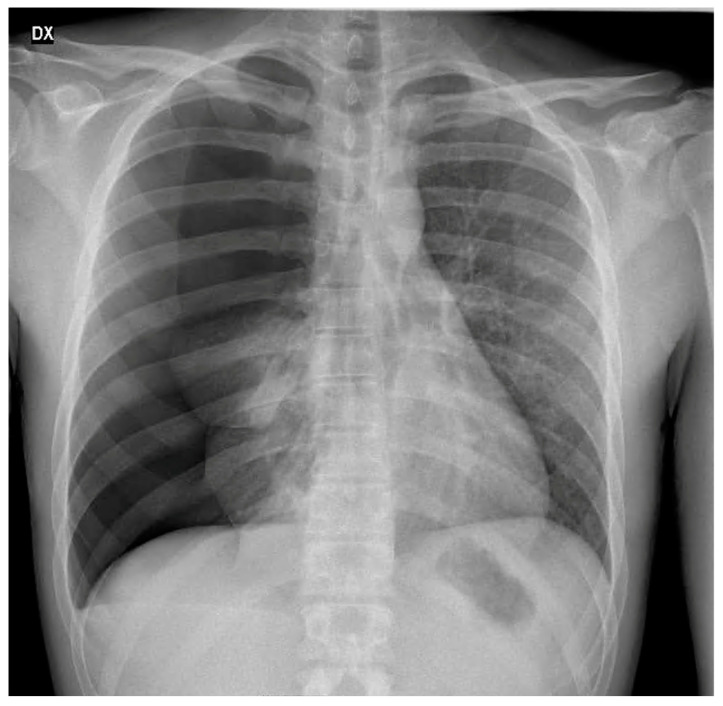
X-ray showing massive right idiopathic pneumothorax.

A 29-year-old man, an active tobacco smoker, and occasional marijuana user reported right shoulder chest pain and the worsening of respiratory symptoms over the previous 5 days. Due to the onset of tachycardia and general discomfort, the patient sought medical attention at the hospital’s emergency room. Vital parameters at admission were SpO_2_ 93% in ambient air and a pulse rate of 102 beats/min with stable arterial blood pressure. The diagnosis of massive right idiopathic pneumothorax was confirmed at chest X-ray (Figure 5) and promptly treated with chest drain placement, performed under fluoroscopy to ensure proper lung expansion.

**Figure 6:**
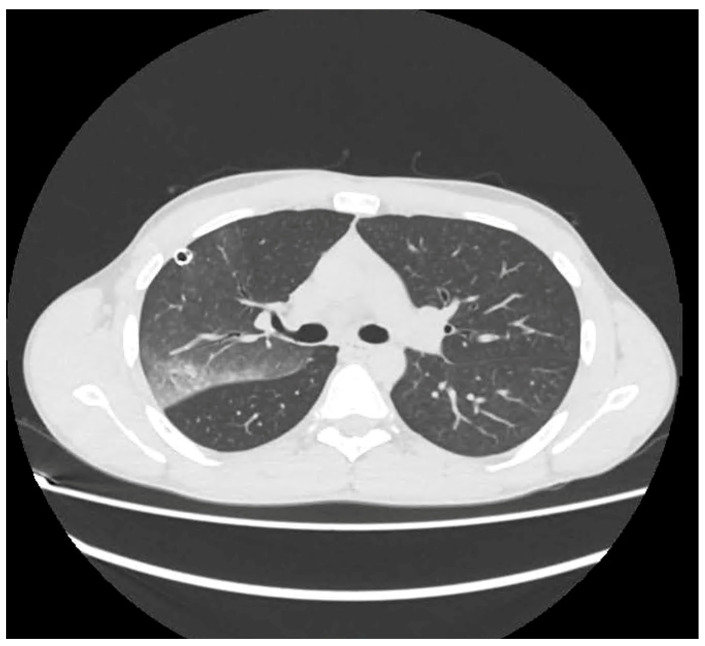
CT scan of right re-expansion pulmonary edema with alveolar filling and a consolidative area with air bronchogram.

After the procedure, the patient complained of an initially dry cough, later accompanied by the expectoration of clear mucus, followed by severe dyspnea, tachypnea, and tachycardia. There was no evidence of significant air leakage from the chest drain. Immediate treatment included O_2_ therapy with a Venturi Mask with 35% FiO_2_, diuretics, bronchodilators, and steroids. Blood gas analysis was performed showing pH 7.49; PaCO_2_ = 29 mmHg; PaO_2_ = 58 mmHg; bicarbonate 26 mM; and SaO_2_ 91%. A CT scan confirmed right re-expansion pulmonary edema with alveolar filling and a consolidative area with an air bronchogram (Figure 6).

The patient was then supported with HFNO (flow rate 60 L/min, FiO_2_ 40%). Radiological controls after 3 days showed the resolution of re-expansion pulmonary edema. Six days later, the patient underwent thoracoscopic apicectomy and mechanical pleurodesis due to persistent pneumothorax. The postoperative course was smooth, and the patient was discharged four days after surgery.

## 4. Literature Review

### 4.1. Background

Re-expansion pulmonary edema represents a complication with multiple causative factors that have not been fully elucidated, and there is ongoing controversy surrounding data related to the incidence, predisposing factors, and mortality rate. Some authors conducted comprehensive reviews on the incidence of re-expansion pulmonary edema. For instance, Echevarria et al. [11], examining papers dated from 1950 to January 2008, including both pneumothorax and pleural effusions, found that out of the 233 papers, 13 provided insights into the occurrence of re-expansion pulmonary edema, citing an incidence ranging from 0% to 1%. In another study over 8 years, Rozenman et al. [12] documented their experience with 180 patients who underwent 320 episodes of pneumothorax, identifying only 3 cases of re-expansion pulmonary edema. These informative studies, along with others [6,7], collectively indicate that re-expansion pulmonary edema is generally an infrequent occurrence. However, considering asymptomatic or paucisymptomatic cases, the actual incidence should be significantly higher than the 1% mentioned in these studies.

### 4.2. Pathogenesis and Risk Factors

The pathogenesis of re-expansion pulmonary edema is still unknown and is probably multifactorial, prompting several authors to investigate its potential risk factors.

Various hypotheses have been proposed, in particular, in the etiological process of re-expansion pulmonary edema. They involve the chronicity of collapse, the technique of re-expansion, and modifications of pulmonary vascular permeability. Nevertheless, none of them are considered essential for predicting the occurrence of re-expansion pulmonary edema. Although re-expansion pulmonary edema typically manifests in cases of chronic lung collapse and rapid lung expansion following the removal of substantial amounts of air or fluid, it is not a universal rule. The majority of studies indicate, as in our series, that patients with re-expansion pulmonary edema experienced a pneumothorax lasting 3 days or more, as reported by Mahfood et al. [7]. An animal study conducted by Miller et al. [13] supports the hypothesis that the length of pneumothorax’s onset and the application of suction after drainage could be considered risk factors for the development of re-expansion pulmonary edema. Miller demonstrated in Rhesus monkeys undergoing unilateral pneumothorax, maintained for 3 days, that rapid re-expansion of the lung, coupled with suction application, led to significant acute pulmonary edema on the same side within 2 h, compared to the group of monkeys undergoing only water seal drainage. In another group of monkeys, pulmonary edema did not occur when a pneumothorax was drained within 3 days with the application of suction [13]. The role of pneumothorax size as a risk factor for re-expansion edema is still a debated issue [14], with some authors suggesting its significance, while other studies do not establish a clear association. It is possible that patients with milder degrees of lung collapse have a longer duration of symptoms; conversely, those with more severe lung collapse exhibit a shorter duration of symptoms and receive earlier treatment [7]. Similar conditions can be observed after the evacuation of large amounts of fluid during thoracentesis. The rapid aspiration of more than 2 L of fluid is associated with re-expansion pulmonary edema [7]. Guidelines from prominent medical societies recommend removing less than 1.5 L of pleural fluid at a time to avoid high negative intrapleural pressures during therapeutic thoracentesis [15]. On the other hand, because the safe aspiration of much larger volumes has been documented without complications with a low rate of re-expansion pulmonary edema, some authors [6] have questioned the recommendation that thoracentesis should be terminated after removing 1 L of fluid, suggesting that large effusions should be drained completely. Indeed, the incidence of re-expansion pulmonary edema after large-volume thoracentesis appears to be unrelated to the amount of fluid removed, pleural pressures, and pleural elastance. The ultimate evidence suggests that large-volume thoracentesis can be safely conducted by incorporating pleural manometry and ultrasound guidance. This approach aids in assessing the patient’s response to fluid removal and monitoring the drop in pleural pressure [16,17]. Other hypotheses support the idea that changes in vascular permeability may play a role in this process. Re-expansion pulmonary edema may result directly from the traction exerted on blood vessels during rapid lung re-expansion, leading to the increased permeability of damaged pulmonary blood vessels [18]. Alternatively, some authors support the effect of ischemia/reperfusion injury of the genesis of re-expansion pulmonary edema. The prolonged compression of pulmonary blood vessels due to pneumothorax or pleural effusion likely triggers hypoxic vasoconstriction of the tissue. The re-expansion of the parenchyma due to extensive drainage results in the reperfusion injury of the collapsed lung, leading to a substantial generation of reactive oxygen species (ROS). These ROS activate the cascade of ischemia/reperfusion injury, causing severe impairment to cell membranes and simultaneously increasing the permeability of the vessel wall. Consequently, macro-molecules extravasate into the alveolar space. It is undeniable that additional factors, including the amount of effusion, duration of collapse, patient age, oxidative stress, etc., also contribute to the complete manifestation of the syndrome [18,19].

### 4.3. Clinical and Radiological Features

The clinical presentation of re-expansion pulmonary edema can vary widely, ranging from cases with a positive radiological finding but clinically asymptomatic, to severe forms causing cardiorespiratory failure and circulatory shock. In a significant number of cases, re-expansion pulmonary edema is an asymptomatic or self-limiting condition that does not require any intervention. Patients with symptomatic re-expansion pulmonary edema typically experience rapidly progressive dyspnea and tachypnea, usually occurring within 1 h after lung expansion intercostal drainage. Additional symptoms may include a productive cough, tachycardia, hypotension, fever, chest pain, nausea, and vomiting [20]. The largest proportion of patients develop re-expansion pulmonary edema within 2 h of lung expansion, with all cases manifesting within 24 h [7]. The early recognition of the clinical picture of re-expansion pulmonary edema is important for initiating prompt management and allowing for a favorable outcome. However, in some instances, signs and symptoms may worsen despite treatment, leading to fatalities. Mortality has been reported in several papers. In the earliest studies about re-expansion pulmonary edema, the mortality rate was higher than 20% [7]; however, more recent studies have found this value to be significantly lower, indicating potential overestimation in earlier assessments [14]. These findings suggest that re-expansion pulmonary edema is a more common, transient, and generally benign phenomenon than was previously thought. Radiographic features show a unilateral alveolar filling pattern visible within 2–4 h after re-expansion. Typically, in the case of pneumothorax, the first control performed immediately after chest drain placement is normal, and radiological signs of edema appear one hour later. The edema often progresses over 48 h toward the complete white-out of a hemithorax on the chest X-ray (Figure 1) with persistence for 4–5 days before resolving in a few days, leaving no lasting radiographic abnormalities. On the CT scan, the most common findings include ipsilateral consolidation, ground-glass opacity, intralobular interstitial thickening, interlobular septal and bronchovascular bundles thickening [21] (Figure 2A,B).

### 4.4. Management

In the case of symptomatic re-expansion pulmonary edema, management should ensure cardiovascular and respiratory monitoring and provide oxygen therapy. In fact, re-expansion edema, resulting from alveolar filling with fluid, often presents with severe hypoxia. Typically, the patient shows normal or reduced levels of carbon dioxide and an alkaline pH due to compensatory hyperventilation. From a blood gas analysis perspective, the picture may mimic a pulmonary embolism, necessitating consideration in the differential diagnosis. Less commonly, some cases progress to respiratory acidosis, which remains severe despite the correction of hypoxemia [22]. Although the use of diuretics and steroids has been reported, limited evidence exists to support their efficacy [15]. HFNO therapy has been employed in treating re-expansion pulmonary edema, and some clinical evidence supports its use. It provides better oxygenation, generating positive pressure at the end of expiration (PEEP), improving the inspired oxygen fraction, and washing out and reducing pharyngeal dead space and respiratory work. Moreover, HFNO therapy does not interfere with mucociliary clearance [23,24]. The clinical course of re-expansion edema is highly variable, and only rare cases require the use of mechanical ventilation or result in cardiocirculatory failure. This last point is particularly frequent in patients undergoing thoracentesis and affected by pre-existing cardiac comorbidities. Re-expansion edema following pneumothorax, as mentioned earlier, often affects young individuals and causes acute heart failure in a smaller percentage of cases. The choice of timing for non-invasive or invasive ventilation varies widely in the literature, and the use of ventilation during pneumothorax remains a matter of debate. Although the recent use of HFNO has certainly reduced the need for both non-invasive and invasive ventilation, CPAP has been successfully used in several cases in which simple oxygen support is not sufficient [25]. In those cases, the risk of pneumothorax or tension pneumothorax was minimal once a chest drainage had been placed. However, careful consideration is warranted when using CPAP to avoid potential complications, such as open pneumothorax or tension pneumothorax, especially in cases where a small-bore chest tube is placed or in the presence of bullous emphysema. Generally, the use of CPAP is able to restore endoalveolar pressure and achieve the resolution of the clinical picture in almost all cases, and intubation is required only in a few instances in which respiratory acidosis worsens despite the correction of hypoxemia [22,26]. Exceptionally, conventional ventilator therapy can exacerbate ventilation–perfusion mismatch. This is due to the affected lung being in a state of hyperperfusion and poor compliance, while the unaffected lung experiences hypoperfusion but comparatively better compliance. These cases have been successfully treated with alternative methods, such as a double-lumen endobronchial tube [27] with asynchronous differential lung ventilation for 48 h [27] or using selective high-frequency jet ventilation [28].

## 5. Conclusions

In summary, re-expansion pulmonary edema represents a rare multifactorial and complex clinical entity with an uncertain pathogenesis, though risk factors such as the chronicity of lung collapse and rapid re-expansion techniques have been identified. Its clinical presentation can range from asymptomatic to severe clinical pictures. Management strategies include cautious fluid removal, cardiovascular and respiratory monitoring, and the use of High-Flow Oxygen therapy or CPAP in more severe cases. Recent evidence suggests that re-expansion pulmonary edema may not be as rare or as fatal as historically considered, and with careful management, most patients experience the resolution of symptoms and radiological findings without severe long-term consequences.

## References

[1] Yi E., Park J.E., Chung J.H., Ahn C.B., Chung E., Noh O.K., Lee S. (2023). Trends in Recurrence of Primary Spontaneous Pneumothorax in Young Population after Treatment for First Episode Based on a Nationwide Population Data. Sci. Rep..

[2] Pogorelić Z., Gudelj R., Bjelanović D., Jukić M., Elezović Baloević S., Glumac S., Furlan D. (2020). Management of the Pediatric Spontaneous Pneumothorax: The Role of Video-Assisted Thoracoscopic Surgery. J. Laparoendosc. Adv. Surg. Tech. A.

[3] Hippolyte Armand P. (1853). Considérations Cliniques Sur la Thoracentèse. These de Paris.

[4] Riesman D. (1902). Albuminous expectoration following thoracocentesis. Am. J. Med. Sci..

[5] Hartley P.H. (1905). Albuminous expectoration following para-centesis of the chest. St Bartholomew’s Hosp. J..

[6] Feller-Kopman D., Berkowitz D., Boiselle P., Ernst A. (2007). Large-volume thoracentesis and the risk of reexpansion pulmonary edema. Ann. Thorac. Surg..

[7] Mahfood S., Hix W.R., Aaron B.L., Blaes P., Watson D.C. (1988). Reexpansion pulmonary edema. Ann. Thorac. Surg..

[8] Matsuura Y., Nomimura T., Murakami H., Matsushima T., Kakehashi M., Kajihara H. (1991). Clinical analysis of reexpansion pulmonary edema. Chest.

[9] Buczko G.B., Grossman R.F., Goldberg M. (1981). Re-expansion pulmonary edema: Evidence for increased capillary permeability. Can. Med. Assoc. J..

[10] Sprung C.L., Loewenherz J.W., Baier H., Hauser M.J. (1981). Evidence for increased permeability in reexpansion pulmonary edema. Am. J. Med..

[11] Echevarria C., Twomey D., Dunning J., Chanda B. (2008). Does re-expansion pulmonary oedema exist?. Interact. Cardiovasc. Thorac. Surg..

[12] Rozenman J., Yellin A., Simansky D.A., Shiner R.J. (1996). Re-expansion pulmonary oedema following spontaneous pneumothorax. Respir. Med..

[13] Miller W.C., Toon R., Palat H., Lacroix J. (1973). Experimental pulmonary edema following re-expansion of pneumothorax. Am. Rev. Respir. Dis..

[14] Taira N., Kawabata T., Ichi T., Yohena T., Kawasaki H., Ishikawa K. (2014). An analysis of and new risk factors for reexpansion pulmonary edema following spontaneous pneumothorax. J. Thorac. Dis..

[15] Havelock T., Teoh R., Laws D., Gleeson F. (2010). Pleural procedures and thoracic ultrasound: British Thoracic Society Pleural Disease Guideline 2010. Thorax.

[16] Hassaballa A.S., Mostafa A., Hikal T. (2023). Pleural manometry during thoracocentesis in patients with malignant pleural effusion: A randomized controlled trial. Can. J. Respir. Ther..

[17] Feller-Kopman D.J. (2018). Management of Malignant Pleural Effusions. An Official ATS/STS/STR Clinical Practice Guideline. Am. J. Respir. Crit. Care Med..

[18] Sohara Y. (2008). Reexpansion pulmonary edema. Ann. Thorac. Cardiovasc. Surg. Off. J. Assoc. Thorac. Cardiovasc. Surg. Asia.

[19] Sugiyama Y., Shimizu F., Shimizu S., Urasawa M., Tanaka S., Kawamata M. (2015). Severe Re-expansion Pulmonary Edema Induced by One-Lung Ventilation. Respir. Care.

[20] Neustein S.M. (2007). Reexpansion pulmonary edema. J. Cardiothorac. Vasc. Anesth..

[21] Tarver R.D., Broderick L.S., Conces D.J. (1996). Reexpansion pulmonary edema. J. Thorac. Imaging.

[22] Cho S.R., Lee J.S., Kim M.S. (2005). New treatment method for reexpansion pulmonary edema: Differential lung ventilation. Ann. Thorac. Surg..

[23] Lee S.K., Son B.S., Son J., Lee S.E., Yeo H.J., Kim D.H. (2019). High-flow oxygen therapy for treating re-expansion pulmonary edema. Ann. Transl. Med..

[24] Fontoura A., Fernandes F.R., Oliveira M., Santos P., Trindade E.S.L.P. (2021). High-flow nasal oxygen in re-expansion pulmonary oedema. Pulmonology.

[25] Tariq S.M., Sadaf T. (2006). Images in clinical medicine. Reexpansion pulmonary edema after treatment of pneumothorax. N. Engl. J. Med..

[26] La Via L., Sanfilippo F., Cuttone G., Dezio V., Falcone M., Brancati S., Crimi C., Astuto M. (2022). Use of Ketamine in Patients with Refractory Severe Asthma Exacerbations: Systematic Review of Prospective Studies. Eur. J. Clin. Pharmacol..

[27] Solidoro P., Corbetta L., Patrucco F., Sorbello M., Piccioni F., D’Amato L., Renda T., Petrini F. (2019). Competences in bronchoscopy for Intensive Care Unit, anesthesiology, thoracic surgery and lung transplantation. Panminerva Medica.

[28] Wake M., Sanagawa Y., Okamoto Y. (2000). A case of anesthetic management for re-expansion pulmonary edema of the dependent lung saved by superimposed HFJV during one lung ventilation for the thoracoscopic operation associated with bilateral pneumothorax. Masui Jpn. J. Anesthesiol..

